# A98 ASSOCIATIONS BETWEEN DISTANCE TO CLINIC AND PATIENT CHARACTERISTICS FOR YOUNG ADULTS WITH IBD AT THE TIME OF PEDIATRIC TO ADULT TRANSFER OF CARE

**DOI:** 10.1093/jcag/gwac036.098

**Published:** 2023-03-07

**Authors:** A Bihari, K Kroeker, E Wine

**Affiliations:** University of Alberta, Edmonton, Canada

## Abstract

**Background:**

Patients with inflammatory bowel disease (IBD) in childhood are a vulnerable population. Compared to patients diagnosed in adulthood, they may present more often with extensive disease, delayed growth, and more frequently have perianal disease. Eventually, these patients will transfer care from the pediatric to the adult system, which has been associated with medication nonadherence and increase in health care utilization. Within the literature, travel distance has been cited as a barrier to accessing health care; however, understanding its’ impact on this vulnerable patient population has not been well-established.

**Purpose:**

The study objective was to characterize differences between patients transferring from pediatric to adult care based on distance from an IBD center. To achieve this, the study aims to measure associations between distance to clinic and smoking status, anxiety and depression, history of surgery, and biologics at the time of first appointment in adult care.

**Method:**

A retrospective cross-sectional study is ongoing using electronic medical charts for patients who transferred from pediatric to adult care from January 1, 2014 – October 4, 2022. Transfer was defined as the patient’s first appointment in adult care at the University of Alberta’s IBD clinic. Distance was measured by driving distance from the patient’s postal code to the postal code of the IBD clinic. Distance was categorized as being <50km from IBD clinic and >50km. Binary outcome variables collected at time of transfer included reported biologic use, anxiety and depression, smoking status, and history of surgeries. Descriptive and inferential statistics were used to analyze data.

**Result(s):**

Of the 185 electronic medical charts were reviewed, 46 (24.9%) patients lived >50km from clinic. The median age at diagnosis for the >50km group was 14.5 (IQR: 15.9-13.6), 41% were female sex, 54% had Crohn disease, 41% had ulcerative colitis (UC), and 26% had completed a fecal calprotectin within the last 6 months. The median age at diagnosis of the 139 patients living <50km from clinic was 14.3 (IQR:15.9-12.5), 45% were female sex, 60% had Crohn disease, 32% had UC, and 59% had completed a fecal calprotectin. Of those who had a fecal calprotectin, in group 1, 26% had a result over >250 ug/g, compared to 60% in the reference group. Univariate analysis (Table 1) showed that those living further than 50 km were more likely than those closer to engage in daily smoking (OR~4.7). Weaker and lack of associations were seen with anxiety and depression (OR~1); being on biologics (OR~0.7); history of surgical intervention (OR~2.2).

**Image:**

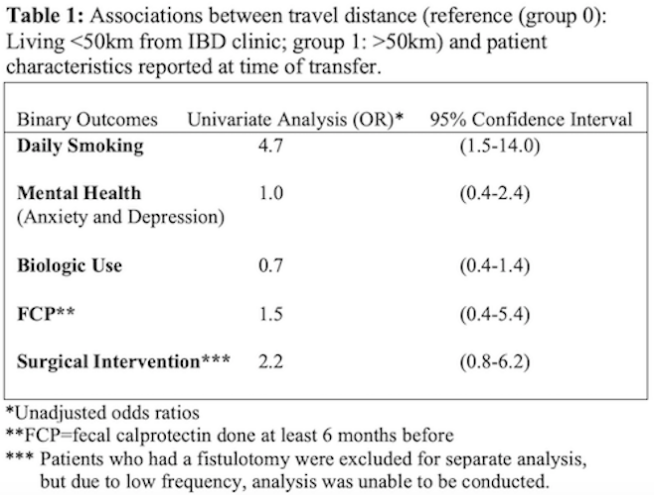

**Conclusion(s):**

When smoking status was reported, patients > 50km away from clinic were 4.7 times more likely to engage in daily smoking at time of transfer compared to those patients within 50km from the clinic. By acknowledging and understanding potential differences and similarities between patients characterized by geographical location, we can use this research to inform personalized care plans.

**Disclosure of Interest:**

None Declared

